# Comparison of postpartum family planning uptake between primiparous and multiparous women in Webuye County Hospital, Kenya

**DOI:** 10.4102/safp.v62i1.5093

**Published:** 2020-08-13

**Authors:** Henry O. Owuor

**Affiliations:** 1 Department of Family Medicine, School of Medicine, Moi University, Eldoret, Kenya

**Keywords:** Family planning; postpartum family planning, primiparity, multiparity, reproductive health, rural medicine

## Abstract

**Background:**

Postpartum family planning (PPFP) is associated with health, social and economic benefits to a woman and her family. Its uptake, particularly of the more effective, long-acting reversible contraceptives (LARCs), is low. The role of parity in PPFP uptake is inconclusive. The aim of this study was to compare the uptake of PPFP and LARCs between primiparous and multiparous women accompanying their children for the first measles vaccine, which is at 9 months after delivery, in Webuye County Hospital, Kenya.

**Methods:**

This was a cross-sectional study. Study participants were recruited using a systematic random sampling method and data were collected using a pretested, structured, interviewer-administered questionnaire. The collected data were analysed using an independent *t*-test to compare PPFP uptake between primiparous and multiparous women, whereas chi-square tests (for categorical data) and independent *t*-tests (for numerical data) were used to compare the various socio-demographic characteristics and occurrence of various predictors of PPFP uptake between the two groups of postpartum women. Factors that were significantly different between the two groups were controlled for using logistic regression.

**Results:**

There was a significant difference on PPFP uptake (22.0%; 95% CI: 11.8–32.3; *p* < 0.001), but none on LARC use (OR = 0.88; 95% CI: 0.46–1.66) between the two groups of women. The unadjusted and adjusted OR for the effect of parity on FP uptake was 3.48 (95% CI: 1.88–6.42) and 2.32 (95% CI: 1.15–4.67), respectively.

**Conclusion:**

There is a significant difference in the uptake of PPFP, but not LARCs, between primiparous women and multiparous women accompanying their children for the 9-month measles vaccine in Webuye County Hospital. Primiparous women are less likely to initiate the use of PPFP compared to their multiparous counterparts.

## Introduction

The foremost benefit of family planning (FP) is the health benefits that accrue to the woman and her baby.^1^ These health benefits are more marked in the period following delivery because a subsequent pregnancy following childbirth holds the greatest risk for the woman and her baby.^2^ On the flip side, however, this period of vulnerability is always associated with a low uptake of FP.^1^ Family planning during this period is christened postpartum FP (PPFP) and is the prevention of unwanted and closely spaced pregnancies during the first 12 months after delivery.^1,3^ A high PPFP uptake results in a reduced risk of the following: miscarriage, low birth weight, neonatal death, maternal death, preterm birth, anaemia and premature rupture of membranes.^3,4^

Even though a subsequent pregnancy holds the greatest risk for mother and baby in the postpartum period, this phase offers numerous opportunities for the provision of PPFP as there are numerous contacts with either the healthcare provider or the health system in pursuit of postnatal care.^5^ In fact, attempts have been made the world over to integrate PPFP into maternal, newborn and child health services so that the contraceptive needs of postpartum women and couples are effectively met.^6^ Postpartum FP has therefore become a key marker for high quality and integrated health services.^2^ Health systems must not miss these myriad opportunities to offer women PPFP information and services.^5^

Long-acting reversible contraceptives (LARCs) – that is, intrauterine devices and progesterone implants – are the most effective contraceptives and in efficacy are comparable to permanent sterilisation methods.^7^ Long-acting reversible contraceptives are recommended as first-line contraceptive choices for all women, including those with or without children of any age, adolescents, and women above 40 years of age.^8,9^ Despite their safety, efficacy, reversibility, acceptability, fewer contraindications and being more long-lasting, the use of LARCs is still low – their use is at 13% and 5% among married women using family planning in the world and in Africa, respectively.^10^

The identification of barriers and facilitators of PPFP uptake is useful in modifying practice among healthcare workers who provide these services.^11^ Various studies have been conducted to elucidate these factors. These include the pattern of breastfeeding, occupation, preferred number of children, place of residence, mode of delivery, partner’s education level, socioeconomic status and postnatal care, among others.^12,13,14^ In western Kenya, a study in the same population of this study’s interest found that the only significant finding was women living with their sexual partners. These women were less likely to use PPFP than those not living with their partner.^11^

The role of parity on PPFP uptake has not been conclusive. Some studies have shown that multiparity is associated with contraceptive use.^15,16,17^ It has been postulated that couples usually start using FP methods after they have reached their desired family size. This often corresponds to higher parities as they seek to limit or space childbirth.^15,17^ Other studies have found no significant association between parity and modern contraceptive use in the postpartum period.^5^

The aim of this study was to add to the available evidence on the role of parity on PPFP uptake by comparing the uptake of PPFP and LARCs between the primiparous and the multiparous women accompanying their children for the first measles vaccine in Webuye County Hospital, Kenya.

## Research methods and design

This was a cross-sectional study that targeted all postpartum women accompanying their children for the 9-month measles vaccine at the hospital’s maternal child health clinic.

Webuye County Hospital is in Bungoma County in western Kenya. According to its annual operating plan for the 2018–2019 financial year, it had a catchment population of 98 494 people. It catered for the needs of 21 669 women of reproductive age (15–49 years) in the 2018–2019 financial year.

The formula for estimating single population proportion was used to arrive at a sample size of 259 participants (an assumption of 95% confidence level, margin of error of 5% and expected prevalence of PPFP in Kenya of 51.1% had been made). Study participants were recruited using systematic random sampling. Data were collected using a pretested, structured, interviewer-administered questionnaire.

The filled questionnaires were checked daily for completeness, coded and data compiled in a Microsoft Excel database. The database was cleaned and the data exported to Statistical Package for Social Sciences-IBM version 20 for analysis. To compare PPFP uptake between primiparous and multiparous women, an independent *t*-test was utilised. To compare the various socio-demographic characteristics and occurrence of various predictors of PPFP uptake between the two groups of postpartum women, chi-square tests (for categorical data) and independent *t*-tests (for numerical data) were used. Logistic regression was undertaken to control for the effects of other confounding factors that were found to be significantly different between the two groups during comparison.

The study was approved by the Moi Teaching and Referral Hospital’s Institutional Research and Ethics Committee. Administrative authorisations were obtained from the medical superintendent of Webuye Hospital, while informed consent was obtained from the study participants.

### Ethical consideration

The study was approved by the Moi Teaching and Referral Hospital’s Institutional Research and Ethics Committee and was assigned a formal approval number FAN: IREC 1663 on 30 June 2016.

## Results

The study recruited 258 study participants. Of these, 92 were primiparous and 166 were multiparous.

The primiparous women were younger and had more years of formal education. They formed more than four fifths of the women who were single. More than 90% of these women wanted an additional child. Only a 10th of them had experience with any form of contraception before the last pregnancy; of these, none had used a LARC. These women also had more of the unplanned pregnancies as they did not use FP (see Table 1).

**TABLE 1:** Demographic characteristics.

Variable	Primiparous			Multiparous			Test-statistic			*p*
	Mean ± SD	*n*	%	Mean ± SD	*n*	%	*t*	*X* ^2^	*df*	
Age (years)	22.23 ± 0.70	-	-	29.64 ± 0.79	-	-	−12.418	35.719	2	< 0.001
**Marital status**	-	-	-	-	-	-	-	-	-	< 0.001
Married	-	65	70.6	-	159	95.8	-	-	-	-
Single	-	26	28.3	-	5	3.0	-	-	-	-
Separated	-	1	1.1	-	2	1.2	-	-	-	-
Years of formal education	11.23 ± 0.54	-	-	9.75 ± 0.54	-	-	3.549	44.291	2	< 0.001
**Plan for future child**	-	-	-	-	-	-	-	-	-	
No	-	5	5.4	-	74	44.6	-	-	-	< 0.001
Yes	-	84	91.3	-	85	51.2	-	-	-	-
Maybe	-	3	3.3	-	7	4.2	-	-	-	-
Number of children if planning: median (IQR)	-	2	0	-	1	1	-	-	-	-
Duration to wait before next birth (years)	4.24 ± 0.42	-	-	3.72 ± 0.29	-	-	1.998	128.81	1	0.047
**History of FP use**	-	-	-	-	-	-	-	-	-	< 0.001
No	-	82	89.1	-	27	16.3	-	-	-	-
Yes	-	10	10.9	-	139	83.7	-	-	-	-
**FP method use**	-	-	-	-	-	-	-	4.028	2	0.122
Natural	-	0	0.0	-	8	5.8	-	-	-	(LR = 0.032)
Hormonal and barrier	-	10	100.0	-	97	69.8	-	-	-	-
LARCs	-	0	0.0	-	34	24.4	-	-	-	-
**LARC use**	-	-	-	-	-	-	-	3.169	1	0.075
No	-	10	100.0	-	105	75.5	-	-	-	(LR = 0.020)
Yes	-	0	0.0	-	34	24.5	-	-	-	-
**Was pregnancy planned**	-	-	-	-	-	-	-	28.282	1	< 0.001
No	-	52	56.5	-	39	23.5	-	-	-	-
Yes	-	40	43.5	-	127	76.5	-	-	-	-
**Reason for unplanned pregnancy**	-	-	-	-	-	-	-	31.197	2	0.001
Not on FP	-	51	98.1	-	19	48.7	-	-	-	-
FP failure	-	1	1.9	-	14	35.9	-	-	-	-
Other …	-	0	0.0	-	6	15.4	-	-	-	-

*df*, degrees of freedom; FP, family planning; LARC, long-acting reversible contraceptive; *X*^2^, Pearson’s chi-square; LR, likelihood ratio; *t*, student’s test statistic; SD, standard deviation.

There was a significant difference on PPFP uptake (OR = 3.48; 95% CI: 1.88–6.42), but none on LARC use (OR = 0.88; 95% CI: 0.46–1.66) between the two groups of women (see Tables 2 and 3).

**TABLE 2:** Comparison of postpartum family planning uptake using the independent samples *t*-test.

Group statistics	Parity	*N*	Mean	SD	SE of mean
Women on FP	primis	92	0.64	0.482	0.050
	multips	166	0.86	0.347	0.027

SD, standard deviation; SE, standard error; FP, family planning.

**TABLE 3:** Comparison of postpartum family planning uptake using the independent samples *t*-test.

Variable	Levene’s test for equality of variances		*t*-test for equality of means					
	*F*	Sig.	*t*	*df*	Sig. (2-tailed)	Mean diff.	SE of diff.	95% CI
**FP use**								
Equal variances assumed	61.647	< 0.001	−4.233	256	< 0.001	−0.220	0.052	−0.323 to −0.118
Equal variances not assumed	-	-	−3.861	144.029	< 0.001	−0.220	0.057	−0.333 to −0.107

SE, standard error; FP, family planning; Sig., significance; *df*, degree of freedom; diff., difference; CI, confidence interval; *F*, F-statistic/F-value ; *t*, t-statistic.

The factors that were significantly different between the two groups of women include age, marital status, years of formal education, plans for future children, average duration they are willing to wait for the next child, history of FP use, FP method used, FP services at the child welfare clinic, living with the partner in the same house, and discussing FP with the partner (see Table 4).

**TABLE 4:** Predictors of postpartum family planning uptake by parity.

Variable	Primiparous		Multiparous		Test-statistic			*p*
	*n*	%	*n*	%	*t*	*X* ^2^	*df*	
**ANC attendance**	**-**	**-**	**-**	**-**	**-**	0.555	1	0.458
No	0	-	1	-	**-**	**-**	**-**	LR = 0.349
Yes	92	-	165	-	-	**-**	**-**	-
**ANC number: median (IQR)**	**-**	**-**	**-**	**-**	0.774	**-**	**-**	0.440
Median	4	-	4	-	-	**-**	**-**	-
IQR	2	-	2	-	-	**-**	**-**	-
**Place of delivery**	**-**	**-**	**-**	**-**	-	2.089	2	0.352
Public	76	82.6	133	80.1	**-**	**-**	-	-
Private	7	7.6	8	4.8	-	**-**	**-**	-
Other …	9	9.8	25	15.1	-	**-**	**-**	-
**PNC attendance**	**-**	**-**	**-**	**-**	**-**	0.241	1	0.623
No	61	66.3	115	69.3	**-**	**-**	-	-
Yes	31	33.7	51	30.7	-	**-**	**-**	-
**FP services provided at ANC**	**-**	**-**	**-**	**-**	**-**	2.146	1	0.143
No	74	80.4	144	86.7	**-**	**-**	-	-
Yes	18	19.6	22	13.3	-	**-**	**-**	-
**FP services at delivery**	83	90.2	142	85.5	**-**	1.296	1	0.255
No	25	30.1	33	23.2	**-**	**-**	-	-
Yes	58	69.9	109	76.8	-	**-**	**-**	-
**FP services at CWC**	**-**	**-**	**-**	**-**	**-**	7.146	1	0.008
No	15	16.3	10	6.0	**-**	**-**	-	-
Yes	77	83.7	156	94.0	-	**-**	**-**	-
**On PPFP**	**-**	**-**	**-**	**-**	**-**	16.880	1	< 0.001
No	33	35.9	23	13.9	**-**	**-**	-	-
Yes	59	64.1	143	86.1	-	**-**	**-**	-
**LARC use**	**-**	**-**	**-**	**-**	**-**	0.162	1	0.686
Non-LARC	38	64.4	97	67.4	-	-	-	-
LARC	21	35.6	47	32.6	-	-	-	-
**Living with partner**	**-**	**-**	**-**	**-**	-	20.326	1	< 0.001
No	32	34.8	19	11.4	-	-	-	-
Yes	60	65.2	147	88.6	-	-	-	-
**Discussed FP with partner**	**-**	**-**	**-**	**-**	-	19.454	1	< 0.001
No	30	32.6	17	10.2	-	-	-	-
Yes	62	67.4	149	89.8	-	-	-	-

ANC, antenatal clinic; IQR, inter-quartile range; PNC, postnatal clinic; CWC, child welfare clinic; *df*, degrees of freedom; FP, family planning; LARC, long-acting reversible contraceptive; *X*^2^, Pearson’s chi-square; LR, likelihood ratio; *t*, student’s test statistic.

When each of the other factors was controlled for in a logistic regression – that is, used as covariates to parity – the adjusted odds ratios (AOR) for parity remains significant and ranges between 1.78 and 4.094. The factors that remain significant when paired with parity in a logistic regression are marital status (AOR = 2.00; 95% CI: 1.10–3.64; *p* = 0.023); history of FP use (AOR = 0.38; 95% CI: 0.16–0.93; *p* = 0.028); if the pregnancy was planned (AOR = 0.42; 95% CI: 0.22–0.80; *p* = 0.008); whether the woman lives with the partner under one roof (AOR = 0.16; 95% CI: 0.08–0.33; *p* < 0.001); and whether they had discussed FP with the partner (AOR = 0.18; 95% CI: 0.09–0.38; *p* < 0.001).

When a multiple logistic regression was done with these factors as covariates of parity, only living with the partner in the same house (AOR = 0.14; 95% CI: 0.05–0.38; *p* < 0.001) and having discussed FP with the partner (AOR = 0.30; 95% CI: 0.11–0.81; *p* = 0.017) remained significantly associated with the difference in the uptake of FP between the two groups. When a multiple logistic regression was undertaken with these factors as covariates of parity, the AOR for parity became 2.32 (95% CI: 1.15–4.67; *p* = 0.018), which is still significant. Therefore, the unadjusted and adjusted odds of multiparous women having PPFP compared to the primiparous women was 3.48 (95% CI: 1.88–6.42) and 2.32 (95% CI: 1.15–4.67), respectively. The other factors that remained significantly associated with the difference in PPFP uptake were living with the partner in the same house and having discussed FP with the partner.

## Discussion

The study compared the uptake of PPFP and LARCs between primiparous and multiparous women. There was a significant difference on PPFP uptake, but none on LARC use between the two groups of women. The multiparous women were two times more likely to use PPFP than primiparous women.

Parity is the number of live births borne by a woman. This is categorised as either primiparity (one live birth) or multiparity (more than one live birth).^18^ A primiparous woman is therefore one who has had one live birth, whereas a multiparous woman has had more than one live birth. The reason given for the higher PPFP uptake among multiparous women is that when a woman has had a higher number of children, her desire to either space or limit childbearing is usually stronger.^15,19^ For the same reason, it has been demonstrated in other studies that couples usually delay contraceptive use until after attaining their desired family size.^15^ In this study, this is hinted at by the significant difference in the number of multiparous women compared to primiparous women who do not plan to get pregnant (44.6% vs. 5.4%; *p* < 0.001%). After attaining the desired family size, as with these multiparous women, there is higher contraceptive use. Conversely, only one out of every 10 primiparous women has a history of FP use, and none had ever used a LARC. This reinforces the notion of a stronger desire to limit or space childbearing as the explanation for the difference in FP uptake between the two groups of women. In addition, the primiparous women were more likely to have unplanned pregnancies resulting from the failure to use contraception.

The significant differences in partner support, specifically whether they stay together or have discussed FP options, could be contributive to the differences in PPFP uptake.^11^ The occurrence of these factors was lower among the primiparous women than among the multiparous women.

### Strengths and limitations

Because this study was a cross-sectional study, recall bias and social desirability bias are all inherent weaknesses. These were minimised by assuring the study participants of confidentiality and interviewing them in a private area. The social distance was also minimised. The study was conducted in a single facility, but findings are generalisable to other facilities in the region or to the population in this geographical area, given the high prevalence of the measles vaccine coverage in Bungoma (84.3%).

### Recommendations

Since primiparous women are less likely to initiate the use of PPFP, their education on the benefits of FP has to be planned and emphasised by the healthcare providers during antenatal care as well as postnatal care. Primiparous women were found to have a higher occurrence of unplanned pregnancies because of the non-use of FP, yet they are more educated than the multiparous women. Young women in college and high school should be educated on the benefits and availability of safe and long-acting FP methods. They should be encouraged to initiate the use of FP to prevent unplanned pregnancies.

## Conclusion

There is a significant difference in the uptake of PPFP, but not LARCs, between primiparous women and multiparous women accompanying their children for the 9-month measles vaccine in Webuye County Hospital. Primiparous women are less likely to initiate the use of PPFP compared to their multiparous counterparts. Living with the sexual partner in the same house and discussing FP with the partner are significant predictors of PPFP uptake.
